# Enhanced Vascular Endothelial Growth Factor Gene Expression in Ischaemic Skin of Critical Limb Ischaemia Patients

**DOI:** 10.1155/2012/691528

**Published:** 2012-03-27

**Authors:** Silvia Bleda, Joaquín de Haro, Francisco Acin, César Varela, Leticia Esparza

**Affiliations:** Vascular Surgery and Angiology Department, Getafe University Hospital, Carretera de Toledo km 12,500, 28905 Getafe, Madrid, Spain

## Abstract

*Objectives*. To perform a quantitative analysis of the vascular endothelial growth factor (VEGF) gene transcription in the skin of ischemic legs and provide information for VEGF in the pathogenesis in critical limb ischemia (CLI). *Methods*. Skin biopsies were obtained from 40 patients with CLI. Control samples came from 44 patients with chronic venous disease. VEGF gene expression was analysed using quantitative polymerase chain reaction. *Results*. Patients with CLI had higher skin VEGF expression than control group (RQ: 1.3 ± 0.1 versus 1, *P* = 0.04). *Conclusions*. We found an association between ischemic skin and an elevated VEGF expression in legs from patients with CLI. These data support that the mechanism for VEGF upregulation in hypoxia conditions is intact and acts appropriately in the ischaemic limbs from patients with CLI.

## 1. Introduction

Angiogenesis has been recently involved in the onset and progression of atherosclerosis. The vascular endothelial growth factor (VEGF) is considered a key regulator of angiogenesis. Several researches have demonstrated hypoxia as the major factor in VEGF upregulation [1]. Its expression is dramatically induced by low oxygen tension in endothelial cells [2]. VEGF increases vascular permeability and induces the migration and proliferation of endothelial cells [3]. It has been proved that VEGF promotes an increase in microvessel density and proliferation of *vasa vasorum *in atherosclerotic plaques [4].

Critical limb ischemia (CLI) is the most severe clinical manifestation of peripheral artery disease (PAD). Its World incidence has been estimated to be 500 to 800 per million inhabitants per year [5]. Without timely diagnosis and revascularization, patients with CLI are at risk of devastating complications including loss of limb and life.

Therapeutic angiogenesis has been assessed as an alternative treatment for CLI before the amputation. Thereby, VEGF is one of the most common growth factors used in therapeutic angiogenesis treatment protocols. Disappointingly, the results arose from the clinical trials in therapeutic angiogenesis are not consistent because some of them have not shown a clear effect of this treatment [6]. A likely explanation for these results may lie in the limited knowledge about the complex pathogenesis of CLI and the exact mechanism of action understanding of the therapeutic angiogenesis factors we dispose so far.

On the other hand, recent reports have recently suggested serum VEGF levels to be an indicator of the severity of PAD [7]. CLI is characterized by chronically hypoxic conditions in muscle and epithelial tissues that last for weeks or even months. This hypoxic environment would justify the increased expression of VEGF in these CLI patients. So, whether or not the concentration of VEGF is shown elevated in the CLI patient tissues, the use of any means to increase the levels of VEGF as a CLI therapy should be questioned.

We hypothesize about the possibility that skin in ischemic limbs in patients with CLI, in which hypoxia conditions are critical, could be a source of VEGF. The aim of the current paper is to perform a quantitative analysis of the VEGF gene transcription in the skin of ischemic legs and to provide information on VEGF expression in the pathogenesis of CLI.

## 2. Methods

### 2.1. Patients

84 subjects were included in the present case-control study. The demographic characteristics of the control and CLI patient groups are summarized in Table 1. CLI group patients were recruited at the Vascular Surgery Department at Getafe University Hospital. A patient was included if he met the following inclusion criteria: (1) had symptoms of rest pain or ischemic distal ulcers (Rutherford's classification, grade 4 and 5), (2) had objective signs of distal ischemia—an ankle blood pressure of <60 mm Hg or an ankle-brachial index of <0.5, and (3) was a candidate for infrainguinal autologous vein bypass (TASC C or TASC D lesions [5]) after undergoing angiography. Patients who were decided to undergo endovascular revascularization were not considered to be included.

Forty consecutive patients who met all these criteria underwent below the knee autologous vein bypass, enabling sampling of skin in each patient from the distal part of the calf incision, were included. Twenty-three (57.5%) patients experienced rest pain, and seventeen (42.5%) had ischaemic ulcers affecting the toe. None of the patients had a medically documented history of cancer, arthritis, or other systemic inflammation disease.

The preoperative arteriogram showed chronic total occlusion of superficial femoral artery in 29 out of 40 patients (72.5%). Nine (22.5%) of the patients had chronic total occlusion of popliteal artery and proximal trifurcation vessels, and 2 patients (5%) had a recurrent stenosis after two endovascular interventions.

### 2.2. Control Subjects

44 patients with chronic venous disease, with symptomatic C_2_ grade CEAP classification [8], who were undergoing elective crossectomy with stripping of the great saphenous vein (GSV) served as control subjects. Biopsies were collected from the skin close to the ankle. The control subjects were screened to exclude patients with a medical history of peripheral artery disease or those who demonstrated objective signs of distal ischemia. All the subjects in the control group were demonstrated to show an ankle-brachial index of >0.9.

Controls with a medically history of cancer, arthritis, or other systemic inflammation disease were excluded.

The study was performed according to institutional guidelines and was approved by the Ethics Committee at the Getafe University Hospital. Informed consent was obtained from each patient and control subject before the inclusion in accordance with principles of the Declaration of Helsinki.

### 2.3. Skin Biopsies

Samples were collected during operation. Skin biopsies (2 × 2 mm) were obtained from the distal part of the calf incision in the CLI patients group and from the ankle incision for GSV stripping in controls. Biopsies were immediately introduced into RNAlater solution (Sigma, St. Louis, MO, USA), stored for 24 h at +4°C, and then frozen to −80°C until processing.

### 2.4. VEGF Gene Expression

The samples were later thawed and homogenised using a MagNA Lyser electric homogeniser (Roche, Basel, Switzerland). The RNA was obtained using the commercial RNeasy Fibrous Mini Kit (Qiagen, Hilden, Germany) according to the manufacturer's instructions.

The amount of purified RNA was determined using spectrophotometry at 260 nm in a Nanodrop analyser (ND-100; Nanodrop Technologies, Wilmington, DE, USA). The purity was verified according to the ratio of 260/280 nm measurements, such that values between 1.8 and 2.1 indicated that the quality of the RNA obtained was optimal and suitable for the quantitative real-time polymerase chain reaction (qRT-PCR).

The VEGF gene expression was analysed using qRT-PCR (Real Time PCR 7500 Fast. Version 2.0, Applied Biosystems, Carlsbad, CA, USA). For the qRT-PCR study, 1 *μ*g of total RNA was reverse transcribed to complementary DNA (cDNA) using the commercial High Capacity cDNA Reverse Transcription Kit (Applied Biosystems, Carlsbad, CA, USA). Afterwards, 30 ng of cDNA was used as a mould for the real-time PCR in a final volume of 12 *μ*L which contained TaqMan Master Mix Universal Fast (2x) plus the specific TaqMan (20x) test for the gene studied. Amplification was for 35 cycles of 94°C for 45 s, 58°C for 45 s, and 72°C for 60 s. The gene studied was VEGF (Hs009055_m1) using the following primers: forward 5′-TCAAGGACAGAAGAGACTATAAAATTTGC-3′ and reverse 5′-ACTCCAAACTCCTTCCCCACAT-3′. The 18S gene of ribosomal RNA (Hs99999901_m1) with the following primers: forward 5′-GTAACCCGTTGAACCCCATT-3′ and reverse 5′-CCATCCAATCGGTAGTAGCG-3′ and the house-keeping Cyclophilin gene with primers: forward 5′-AATGCTGGACCAAACACAAA-3′ and reverse 5′-CCTTCTTTCACCTTCCCAAA-3′ were used as an endogenous control.

The relative quantification of the VEGF gene expression was performed using the ΔΔCT comparative method [9]. The results were standardised to the content in the endogenous control. The program calculates the ΔCts and the ΔΔCT with the formulas below:

(1) $$\begin{array}{c} \Delta \text{Ct}=\text{Ct}\_\text{Mean}\;\left(18\text{S}\right)-\text{Ct}\_\text{Mean}\;\left(\text{Target}\right),\\ \Delta \Delta \text{Ct}=\Delta \text{Ct}-\Delta \text{Ct}\_\text{Mean},\\ \text{Gene}\;\text{expression}\;\text{level}=2^{-\text{DDCt}}. \end{array}$$

### 2.5. Statistical Analysis

The Kolmogorov-Smirnov test was used to assess the normality of the distribution of the variables. Comparisons of the VEGF expression were made between the 2 groups using the Mann-Whitney *U* test for independent variables. All calculations were performed using the SPSS program version 15.0, the results were expressed as mean ± standard deviation, and a *P* value < 0.05 in two-tailed analysis was considered statistically significant.

## 3. Results

**Figure 1:**
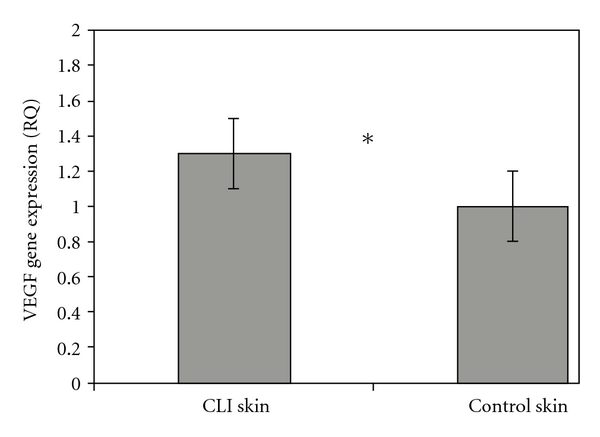
Comparison of the VEGF gene expression for the skin tissue between the CLI group and the control group using the ΔΔCT method. Error bars express the RQ values of 95% confidence interval. *means statistical significance.

Figure 1 shows the data for the VEGF gene expression obtained using the ΔΔCT method after quantitative real-time PCR reaction analysis. This method doubly normalizes quantitative results of the analysis of gene expression by the control genes (18S and cyclophilin), and the tissue was having lower expression of VEGF.

It was shown that VEGF gene was absolutely overexpressed in CLI patient skin compared with cyclophilin gene expression. Cyclophilin is a cyclosporine A cytosolic receptor which is ubiquitously expressed and constantly in the cytosol of all eukaryotic cells and tissues in which it was used as house-keeping gene positive control to demonstrate the relative quantification expression of VEGF in the studied tissues.

An analysis of the relative quantification (RQ) values using the ΔΔCT method was performed for skin tissue between the CLI group and the control group. The results revealed a significant difference in the skin VEGF expression between the two groups. Higher transcription of VEGF gene in the CLI group than in the control group was found (1.3 ± 0.1 versus 1, *P* = 0.04).

## 4. Discussion

Hypoxia, a key in the origin of the etiopathological mechanisms of CLI, is a demonstrated stimulus for VEGF production. The knowledge of the mechanisms of VEGF regulation in CLI is crucial to understand the results arose from new therapeutic angiogenesis approach. The present study has reported that VEGF gene expression was greater in ischaemic skin of CLI patients than in healthy skin of non-arteriosclerotic patients. This data indicate that the atherosclerotic status of CLI cause a higher hypoxia environment which increases VEGF gene expression.

The data we have found of VEGF overexpression in ischaemic skin are consistent with the result previously obtained in animal models of peripheral ischemia [10]. Also, this is in agreement with the results of Choksy et al., who showed an elevation of VEGF expression at distal sites in the ischaemic limbs [11]. Nevertheless, these data differ from those described by Palmer-Kazen et al. [12] who found no association between VEGF concentrations and the ischemic tissue area of the leg in patients with CLI. These inconsistencies may be explained by differences in the kind of tissue analyzed, the product assessed, and the methods used to determine the expression of VEGF. Palmer-Kazen et al. measured the protein concentration of VEGF in biopsies of muscle and skin tissues from calf and thigh by using enzyme-linked immunoassay whereas, in our study, the expression of VEGF gene was determined by mRNA analysis using reliable quantitative real-time polymerase chain reaction.

Otherwise, our results support the hypothesis that ischemia-induced inflammation initiates local growth factor production. In fact, recent data suggest that the inflammation and the VEGF pathways may be related [13]. CRP has been demonstrated to increase VEGF expression in monocytic cells suggesting that CRP could play a role in the angiogenesis process. It is plausible that CLI courses with episodes of hypoxia in which a sharper state of inflammation could be the cause of maintained VEGF production. Moreover, VEGF has been detected in higher levels in serum from patients with CLI than in patients with intermittent claudication [7]. The origin of this circulating VEGF could hypothetically be that beside arterial endothelial cells, others tissues from the chronically ischemic limb as the skin, which is subject to a maintained hypoxia. The present study has proven that VEGF gene expression is greater in ischaemic skin than in healthy skin, observation that suggests that skin in the ischemic leg may be a possible source of this protein.

In addition, our data indicate that the mechanisms for VEGF upregulation in CLI remain intact. These data may question the rationale of VEGF gene therapy.

Nonetheless, the present study has some limitations. First, the difference in the gene expression between groups, although significant, is not high. These data would be stronger if protein quantitative concentration in the tissues samples was assessed. However, we believe this determination could mess the results and confuse the interpretation of these. This assessment should evaluate the protein expression, in parallel to RNA expression. Still, the protein assessment only would had measured the concentration of VEGF in the tissue sample whatever its source. Meanwhile, the objective of this study was, besides, to determine the potential origin of the expression of the VEGF.

Second, the samples of the skin biopsies of the patients group were not obtained from the most ischaemic skin area in these patients. Distal part of the calf incision of the autologous bypass procedure in CLI patients was used as a surrogate of the most ischemic skin area by ethical concerns. To collect a skin sample from a more distal area of the CLI patient legs could have irreversibly damages and increased exposure to amputation to patient limbs. Nevertheless, the study benefited from the homogeneity in the area whence all the samples in the CLI group were obtained, avoiding in this way the potential bias which we could have incurred if the samples came from different skin areas.

In conclusion, this study demonstrates an elevated VEGF expression in the limb ischaemic skin in patients with CLI. These data support that the mechanism for VEGF upregulation in hypoxia environment is intact and acts appropriately in the ischaemic limbs from patients with CLI.

## Figures and Tables

**Table 1:** Clinical and demographic characteristics of CLI and control patients.

	CLI (*N* = 40)	Control (*N* = 44)
Median age	65.7 ± 9.4	47.2 ± 9.9
Males (%)	29 (73.7)	26 (60.1)
Hypertension (%)	34 (84.6)	2 (4.5)
Diabetes mellitus (%)	23 (57)	0 (0)
Dyslipidemia (%)	18 (45)	0 (0)
Acute myocardial Infarction (%)	17 (42.1)	0 (0)
Stroke (%)	4 (10.3)	0 (0)
Renal failure (%)	2 (5.3)	0 (0)
Smoker (%)	25 (63)	2 (4.5)

## References

[1] Shweiki D, Itin A, Soffer D, Keshet E (1992). Vascular endothelial growth factor induced by hypoxia may mediate hypoxia-initiated angiogenesis. *Nature*.

[2] Liu Y, Cox SR, Morita T, Kourembanas S (1995). Hypoxia regulates vascular endothelial growth factor gene expression in endothelial cells: identification of a 5′ enhancer. *Circulation Research*.

[3] Barleon B, Sozzani S, Zhou D, Weich HA, Mantovani A, Marme D (1996). Migration of human monocytes in response to vascular endothelial growth factor (VEGF) is mediated *via* the VEGF receptor flt-1. *Blood*.

[4] Moulton KS, Vakili K, Zurakowski D (2003). Inhibition of plaque neovascularization reduces macrophage accumulation and progression of advanced atherosclerosis. *Proceedings of the National Academy of Sciences of the United States of America*.

[5] Norgren L, Hiatt WR, Dormandy JA, Nehler MR, Harris KA, Fowkes FGR (2007). Inter-society consensus for the management of peripheral arterial disease (TASC II). *Journal of Vascular Surgery*.

[6] de Haro J, Acin F, Lopez-Quintana A, Florez A, Martinez-Aguilar E, Varela C (2009). Meta-analysis of randomized, controlled clinical trials in angiogenesis: gene and cell therapy in peripheral arterial disease. *Heart and Vessels*.

[7] Stehr A, Töpel I, Müller S (2010). VEGF: a surrogate marker for peripheral vascular disease. *European Journal of Vascular and Endovascular Surgery*.

[8] Eklof B, Rutherford RB, Bergan JJ (2004). Revision of the CEAP classification for chronic venous disorders: consensus statement. *Journal of Vascular Surgery*.

[9] Livak KJ, Schmittgen TD (2001). Analysis of relative gene expression data using real-time quantitative PCR and the 2−ΔΔCT method. *Methods*.

[10] Luo F, Wariaro D, Lundberg G, Blegen H, Wahlberg E (2002). Vascular growth factor expression in a rat model of severe limb ischemia. *Journal of Surgical Research*.

[11] Choksy S, Pockley AG, Wajeh YE, Chan P (2004). VEGF and VEGF receptor expression in human chronic critical limb ischaemia. *European Journal of Vascular and Endovascular Surgery*.

[12] Palmer-Kazen U, Wariaro D, Luo F, Wahlberg E (2004). Vascular endothelial cell growth factor and fibroblast growth factor 2 expression in patients with critical limb ischemia. *Journal of Vascular Surgery*.

[13] Bello G, Cailotto F, Hanriot D (2008). C-reactive protein (CRP) increases VEGF-A expression in monocytic cells *via* a PI3-kinase and ERK 1/2
signaling dependent pathway. *Atherosclerosis*.

